# A Real-Time End-to-End Framework with a Stacked Model Using Ultrasound Video for Cardiac Septal Defect Decision-Making

**DOI:** 10.3390/jimaging10110280

**Published:** 2024-11-03

**Authors:** Siti Nurmaini, Ria Nova, Ade Iriani Sapitri, Muhammad Naufal Rachmatullah, Bambang Tutuko, Firdaus Firdaus, Annisa Darmawahyuni, Anggun Islami, Satria Mandala, Radiyati Umi Partan, Akhiar Wista Arum, Rio Bastian

**Affiliations:** 1 Intelligent System Research Group, Universitas Sriwijaya, Palembang 30139, Indonesia; adeiriani@ilkom.unsri.ac.id (A.I.S.); naufalrachmatullah@unsri.ac.id (M.N.R.); bambang_tutuko@unsri.ac.id (B.T.); firdaus@unsri.ac.id (F.F.); annisadarmahyuni@unsri.ac.id (A.D.); 09013682429009@student.unsri.ac.id (A.I.); akhiarwistaarum@unsri.ac.id (A.W.A.); 09011282025039@student.unsri.ac.id (R.B.); 2 Department of Pediatric, Cardiology Division, Dr. Mohammad Hoesin Hospital, Palembang 30126, Indonesia; rianova@fk.unsri.ac.id; 3 Human Centric (HUMIC) Engineering, Telkom University, Bandung 40257, Indonesia; satriamandala@telkomuniversity.ac.id; 4 Department of Internal Medicine, Dr. Mohammad Hoesin Hospital, Palembang 30126, Indonesia; radiyati.u.p@fk.unsri.ac.id

**Keywords:** pediatric, cardiac defect, Yolo, end-to-end

## Abstract

Echocardiography is the gold standard for the comprehensive diagnosis of cardiac septal defects (CSDs). Currently, echocardiography diagnosis is primarily based on expert observation, which is laborious and time-consuming. With digitization, deep learning (DL) can be used to improve the efficiency of the diagnosis. This study presents a real-time end-to-end framework tailored for pediatric ultrasound video analysis for CSD decision-making. The framework employs an advanced real-time architecture based on You Only Look Once (Yolo) techniques for CSD decision-making with high accuracy. Leveraging the state of the art with the Yolov8l (large) architecture, the proposed model achieves a robust performance in real-time processes. It can be observed that the experiment yielded a mean average precision (mAP) exceeding 89%, indicating the framework’s effectiveness in accurately diagnosing CSDs from ultrasound (US) videos. The Yolov8l model exhibits precise performance in the real-time testing of pediatric patients from Mohammad Hoesin General Hospital in Palembang, Indonesia. Based on the results of the proposed model using 222 US videos, it exhibits 95.86% accuracy, 96.82% sensitivity, and 98.74% specificity. During real-time testing in the hospital, the model exhibits a 97.17% accuracy, 95.80% sensitivity, and 98.15% specificity; only 3 out of the 53 US videos in the real-time process were diagnosed incorrectly. This comprehensive approach holds promise for enhancing clinical decision-making and improving patient outcomes in pediatric cardiology.

## 1. Introduction

Cardiac septal defects (CSDs) in pediatric patients, ranging from small holes to larger openings in the septum, pose significant challenges in diagnosis and treatment decisions [1,2]. Current approaches often lack a cohesive framework to integrate diverse data sources, clinical expertise, and decision-support tools, leading to variability in patient care and outcomes [2,3]. Developing an end-to-end framework specifically tailored for CSD decision-making is essential to address these challenges comprehensively [3,4]. In constructing such an effective framework, several factors merit consideration, including defect size and location, the anatomical variability of the defect, the quality of ultrasound (US) imaging, and the patient’s overall condition, among others [4].

However, the most crucial consideration, particularly from the perspective of computer science, lies in several key areas [3,4,5,6]: firstly, developing robust algorithms for image analysis and interpretation that prioritize accuracy, reliability, and generalizability across diverse patient populations and imaging protocols; secondly, validating these algorithms using extensive datasets and independent validation cohorts to ensure consistent performance and clinical relevance; and thirdly, addressing challenges associated with variability in image quality, artifacts, and patient factors (such as motion artifacts and body habitus) that could impact diagnostic accuracy. Previous studies to address this issue have focused primarily on tasks such as classification, segmentation, or detection of CSDs or their individual conditions [7]. However, comprehensive research encompassing the entire methodology of CSD decision-making, from initial data processing to the generation of diagnostic decisions concerning patients’ conditions, remains largely unexplored and exceedingly rare. Hence, the effective end-to-end framework for CSD decision-making is pivotal in determining the necessity for and selection of appropriate treatment modalities, which may include intervention or surgery [3,4,8].

Recent advancements in cardiac US imaging have addressed a variety of areas, including the effectiveness of US-guided procedures, progress in pediatric echocardiography, and the incorporation of deep learning (DL) technologies for detecting cardiac diseases [9,10,11,12,13,14]. Notably, frameworks for managing CSDs now integrate DL methods with cardiac US videos, significantly aiding in the diagnosis of atrial septal defects (ASDs), ventricular septal defects (VSDs), and atrioventricular septal defects (AVSDs) [15,16,17]. This end-to-end framework adopts a multidisciplinary approach, taking into account the diverse anatomical presentations of the defect, its impact on the patient’s health, and the range of methodologies available for closure [8]. By doing so, this framework holds the potential to enhance the efficiency, accuracy, and objectivity of CSD detection and grading, particularly in regions where access to specialized physicians is limited. However, despite these advancements, there remains a scarcity of studies on rare CSDs employing an end-to-end framework for decision-making, primarily due to constraints related to the availability of quality data and the absence of experienced echocardiography experts in many primary healthcare facilities [4].

One of the frameworks has been developed and validated for the automated detection and quantification of ASDs based on color Doppler echocardiography [3]. This framework includes a model for the detection of ASD, which provides a probability level for the presence of the defect, a quantification model that automatically locates the endpoints of the atrial septum, and an estimation size of the defect. The performance of the algorithm was assessed by the bias of the measurement of the defect size and septum length, which provided a quantitative index of the degree of concordance between the DL model and expert physicians. Another framework proposed a fully automatic detection system for ASDs, which includes three stages, such as identifying four target echocardiographic views, segmenting the target cardiac structure and detecting candidates for ASD, and the final decision by utilizing the segmentation and detection results [4]. However, they are proposed only for ASD conditions. To our knowledge, no researcher has created an end-to-end framework for decision-making for CSDs (ASDs, VSDs, and AVSDs). This study makes several contributions, outlined as follows;

■ Introducing an end-to-end model designed for facilitating CSD decision-making. This model involves a comprehensive exploration across five cardiac standard views, namely, apical four-chamber, apical five-chamber, parasternal long axis, parasternal short axis, and subcostal.
■ Developing a stacked Yolo model that integrates normal–abnormal classification, view classification, and defect detection to enhance CSD prediction significantly.
■ Proposing a combination of a stacked Yolo model and an inference algorithm for confirmatory diagnostic evaluation and ensuring its capacity in CSD decision-making.
■ Conducting real-time testing to evaluate the proposed model’s performance with subjects in a hospital setting.

## 2. Materials and Methods

The framework is designed to ensure that the most appropriate methodology is chosen for each patient based on the specific characteristics of their defect and condition. The methodology consisted of (i) Data Preparation; (ii) Introducing End-to-End Framework and Model Evaluation; (iii) Platform; and (iv) Ethics. All processes are conducted based on five standard views including apical four-chamber (A4CH), apical five-chamber (A5CH), parasternal long axis (PLAX), parasternal short axis (PSAX), and subcostal (SC). The entire process is outlined in the following subsections.

### 2.1. Data Preparation

The US video-recording dataset, comprising both normal and CSD conditions—including ASDs, VSDs, and AVSD—was obtained from Dr. Mohammad Hoesin General Hospital in Palembang, Indonesia. This dataset includes 222 US videos, divided into training, validation, and unseen datasets, with recordings from 61 patients with ASDs, 79 patients with VSDs, 7 patients with AVSDs, and 75 patients with normal conditions, all of which were analyzed in this study. Each video, recorded on a Philips ultrasound machine, lasts between one and five seconds and varies in size from 30 to 60 megabytes (MB). All US videos were converted to image files (.jpg), resulting in a total of 151,106 images at 800 × 600-pixel resolution. The details of the dataset of the US video recordings and US images are provided in Table 1.

### 2.2. The End-to-End Framework

**Figure 1:**
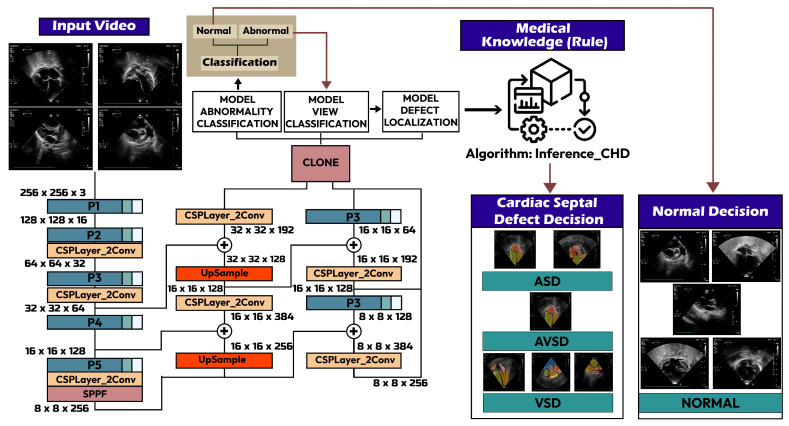
The real-time end-to-end framework.

We propose a real-time end-to-end method with a stacked model encompassing normal–abnormal classification, view classification, defect detection, and a decision-making algorithm based on the medical knowledge of CSDs. Our framework adopts a real-time architecture based on the You Only Look Once (Yolo) algorithm, renowned for its proficiency in object detection (Figure 1) [18]. This algorithm partitions images into a grid and predicts bounding boxes along with class probabilities for these boxes. One notable feature of Yolov8 is its incorporation of a self-attention mechanism within the network’s head. We have developed a comprehensive pipeline to determine the type of CSDs based on the position of the defects. The Yolo architecture was tailored for various tasks executed in a multi-stage process. Unlike conventional models designed primarily for general object detection, our study introduces a stacked algorithm model to provide precise and medically informed decisions. This framework allows the proposed end-to-end model to address distinct aspects of the task, including normal–abnormal classification, standard view classification, defect detection, and clinical decision-making. The process is as follows:

- Normal–abnormal classification: a Yolov8-based classifier is trained to distinguish between normal cardiac anatomy and potential abnormalities.
- Standard view classification: a Yolov8 model is trained to classify images based on standard echocardiographic views, adapting its detection capabilities to recognize specific views that are most relevant for identifying cardiac defects.
- Defect detection: After an abnormality is detected and the view is classified, the model detects specific cardiac defects. Given that defects vary in size and type, Yolov8 is employed to accurately detect and classify these variations, which may present differently across cardiac images.
- Decision-making algorithm: the final step integrates medical knowledge to make a precise decision based on the presence, position, and type of defect, as well as the echocardiographic view.

#### 2.2.1. Normal–Abnormal Classification

Accurate classification of cardiac structures as normal or abnormal (CSDs), is vital for precise decision-making. To achieve this, we propose the Yolov8 architecture, featuring a backbone comprising a cross-stage partial (CSP) Darknet53 architecture where the feature maps into two parts [18]. The first part is applied with convolutional operation, and the second part is concatenated with the previous layer output to enrich the generated feature. In addition, Yolov8 introduces the C2F module, which is an improvement from the C3 module. This combination preserves high-level feature representations to enhance performance outcomes. The architecture employs an anchor-free model with a decoupled head that processes classification, regression, and segmentation tasks independently. This design enables each branch to concentrate on its specific task, thereby enhancing the model’s overall accuracy. In normal–abnormal and view classification tasks, the output layer specifically only used the classification head used during training and testing. The architecture used the cross-entropy loss function combined with the Adam optimizer for training the model hyperparameters. To select the best model, five pre-trained Yolov8 models are compared, including Yolov8n (nano), Yolov8s (small), Yolov8m (medium), Yolov8l (large), and Yolov8x (extra-large) [19,20,21].

#### 2.2.2. Standard View Classification

**Figure 2:**
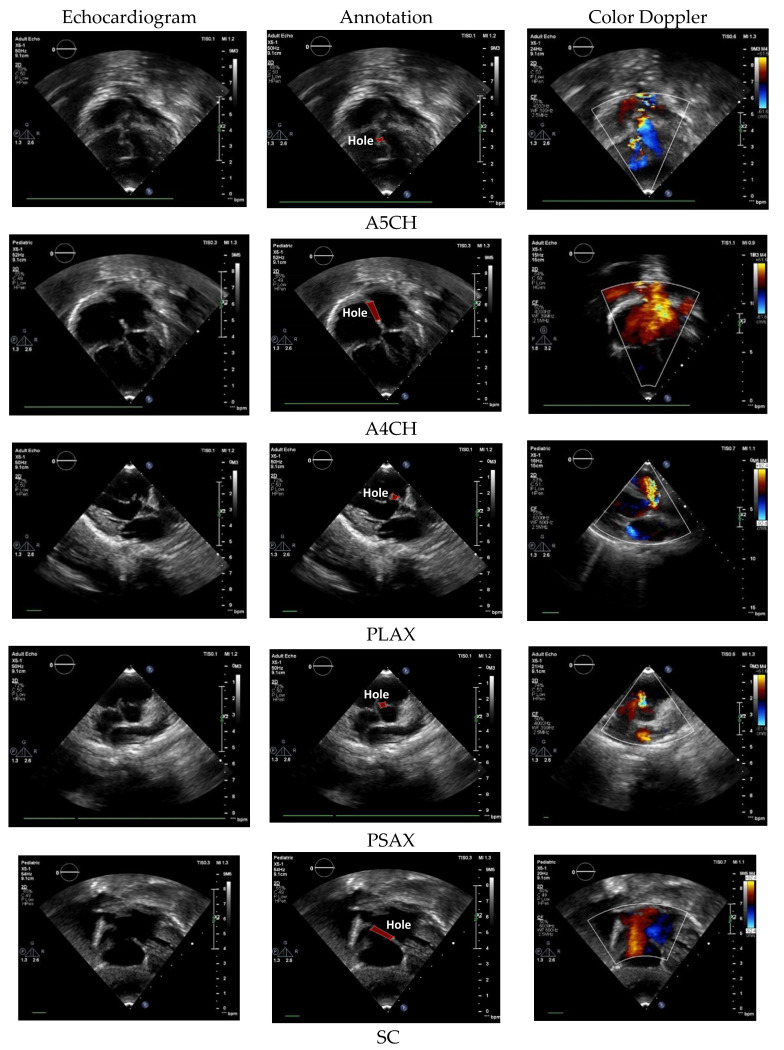
The US standard cardiac view of A4CH, A5CH, PLAX, PSAX, and SC with color Doppler echocardiography.

The main standard cardiac views are A4CH, A5CH, PLAX, PSAX, and SC (Figure 2) [9,10]. A4CH and A5CH in apical view are essential during hemodynamic assessment of the cardiac tissue [11]. A4CH presents the chamber walls of two atria and two ventricles. Different from A5CH, it displays the appearance of the aortic valve and the left ventricular outflow tract [9]. PLAX and PSAX allowed for the measurement of the size of the left atrium, which is used for assessing the valvular function. PSAX provides a cross-sectional view of the heart and allows for an assessment of the left ventricular function [12]. SC provides a similar visualization of the structures seen in apical A4CH, but it is viewed from a different angle [10]. In this study, we also use Doppler US video to ensure the defect condition and provide real-time visualization of blood flow dynamics within the heart. The Yolov8 backbone in this task is similar to normal–abnormal classification. However, the head architecture is increased to five nodes, indicating the class number.

We have formulated a standard view classification algorithm using the Yolo model (Algorithm 1) as follows:
**Algorithm 1.** Inference_View_Echo.
*Input: echocardiograph video, view classifier* *Output: view of echocardiograpy video* *Initialization: assign zero to variaible view_A4CH, view_A5CH, view_PLAX, view_PSAX, view_SC* *BEGIN* *for frame in video do* *view_frame*
*← view_classifier.predict (frame)* *if view_frame = “A4CH” then * *view_A4CH++* *else if view_frame = “A5CH” then*     * view_A5CH++* *    else if view_frame = “PLAX” then* *      view_PLAX++* *    else if view_frame = “PSAX” then* *      view_PSAX++* *    else* *      view_SC++* *view*
*← max (view_A4CH, view_A5CH, view_PLAX, view_PSAX, view_SC)* *return view* *END*


#### 2.2.3. Defect Detection

**Figure 3:**
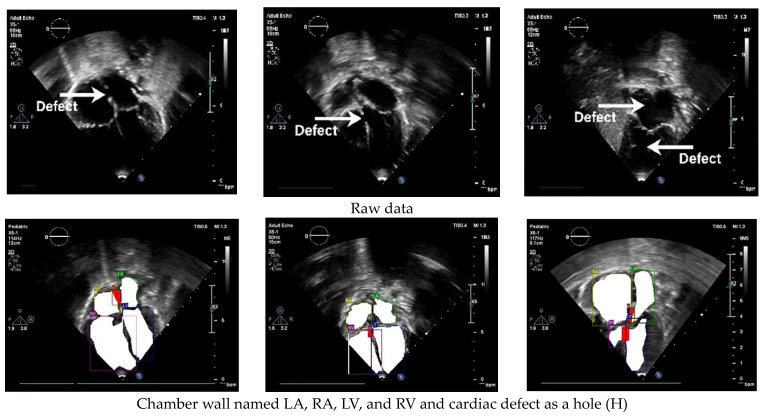
Annotation of chamber wall and cardiac defect.

The standard cardiac view of US video recordings has consisted of A4CH, A5CH, PLAX, PSAX, and SC. Pediatric cardiologists rely on specific views when assessing a patient’s condition. The annotation of the three CSD conditions is presented in Figure 3 using LabelMe 5.3.1 software. The annotated sample results in showcasing the chamber wall area and the cardiac defect area. Atrial or ventricular areas are represented by the white regions, while the red region denotes a cardiac defect area.

#### 2.2.4. CSDs Decision-Making

**Figure 4:**
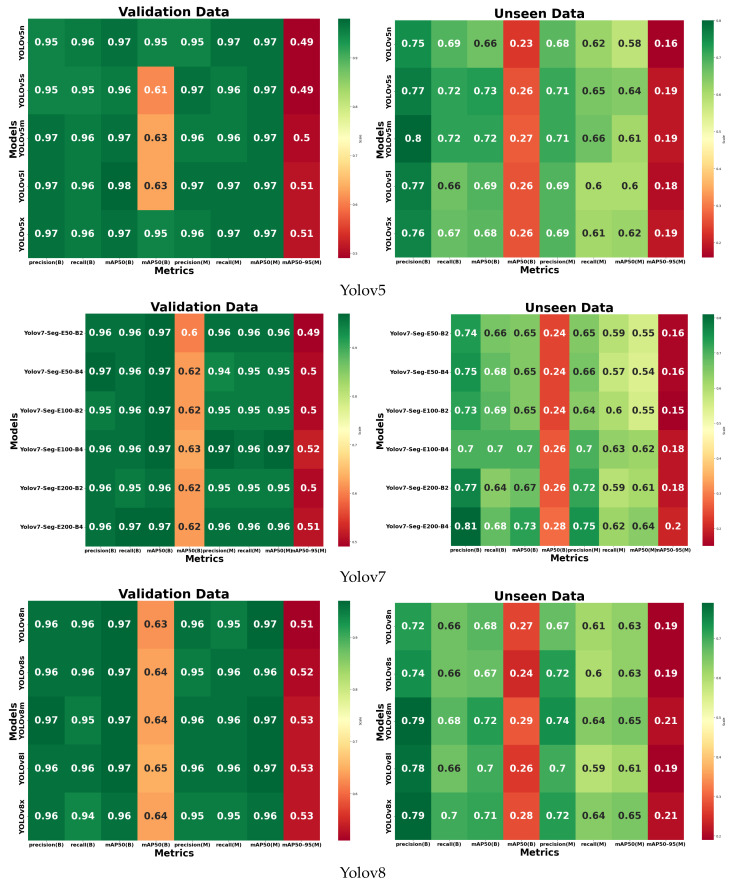
A total of 16 samples for performance comparison with 72 variants of Yolo model for CSD prediction to select the best model.

In the decision-making process for CSDs, this study integrated medical rules with prediction settings for Yolo models (knowledge-based decision-making), as depicted in Figure 4. In order to provide medical rules, we generate an intersection over union value from the hole and chambers. The IoU metric is used to assess the object detection accuracy by measuring the overlap between the actual bounding box and the predicted bounding box. The types of video US CSDs used in this study are outlet perimembranous VSDs, secundum ASDs, and complete/incomplete AVSDs. According to medical standards, the normal heart is assessed using five views (A4CH, A5CH, PLAX, PSAX, and SC), while secundum ASDs are evaluated using two views with A4CH and SC; outlet perimembranous VSDs with three views, A5CH, PLAX, and PSAX; and A complete/incomplete VSDs, only the A4CH view. CSD decision-making based on cardiac standard view is developed based on medical knowledge consisting of the following:

1. ASDs: if the class-predicted cardiac view is A4CH or SC, and the IoU of the hole in the atrium is >0.01 and IoU of the hole in the ventricle is ≤0.01.
2. VSDs: if the class-predicted cardiac view is A5CH or PLAX or PSAX, and IoU of the hole in the atrium is ≤0.01 and IoU of the hole in the ventricle is > 0.01.
3. AVSDs: if the class-predicted cardiac view is A4CH, and IoU of the hole in the atrium is > 0.01 and IoU of the hole in the ventricle is > 0.01.
4. If the knowledge-based decision is not fulfilled, then the predicted class is normal.

Leveraging this medical knowledge, we have formulated a decision-making algorithm (Algorithm 2), as follows:
**Algorithm 2.** Inference_CSDs
*Input: echocardiograph video, view, segment_model_A4CH, segment_model_A5CH, segment_model_PLAX, segment_model_PSAX, segment_model_SC* *Output: echocardiography cardiac septal defects* *BEGIN* *if view = “A4CH” then* *predicted_wall-chamber_and_hole*
*← segment_model_A4CH.predict (video)* *else if view = “A5CH” then* *predicted_ wall-chamber _and_hole*
*← segment_model_A5CH.predict (video)* *else if view = “PLAX” then* *predicted_ wall-chamber _and_hole*
*← segment_model_LA.predict (video)* *else if view = “PSAX” then* *predicted_ wall-chamber _and_hole*
*← segment_model_SA.predict (video)* *else* *predicted_ wall-chamber _and_hole*
*← segment_model_SC.predict (video)* *if “hole” in predicted_ wall-chamber _and_hole then* *iou_hole_atrial*
*← calculate_iou (hole, atrial)* *iou_hole_ventricle*
*← calculate_iou (hole, ventricle)* *if (view = “A4CH” or view = “SC”) and iou_hole_atrial > 0.01 and iou_hole_ventricle*
*≤ 0.01 then* *return “Atrial Septal Defect”* *else if (view = “A5CH” or view = “LA” or view = “SA”) and iou_hole_atrial <= 0.01 and iou_hole_ventricle > 0.01 then* *return “Ventricular Septal Defect”* *else if (view = “A4CH”) and iou_hole_atrial > 0.01 and iou_hole_ventricle > 0.01 then* *return “Atrial Ventricular Septal Defect”* *endif* *else* *return “Normal”* *endif*


### 2.3. Model Evaluation

The proposed classification and detection performance should deliver accurate predictions to provide real value. Equally crucial is evaluating how well the proposed model generalizes on unseen data within the object detection pipeline. In this study, we utilize the mean average precision (mAP) metric to assess the performance of object detection and segmentation systems [16,17,21]. The mAP formula encompasses various sub-metrics, including the confusion matrix, intersection over union (IoU), recall, and precision [16]. The IoU measures the overlap between the predicted bounding box coordinates and the ground-truth box of the real image. A higher IoU indicates closer resemblance between predicted and ground-truth box coordinates. Precision evaluates the model’s ability to generate true positives (TPs) out of all positive predictions, while recall measures TP production out of all predictions. These metrics are computed using an IoU threshold set at 0.5 for object detection tasks. This threshold value may vary based on the model’s confidence threshold, which we set at 0.5 and above [21]. Confidence represents the probability of the existing mass multiplied by the percentage of the IoU between the ground-truth and predicted boxes. It is crucial for refining the model’s predictions and ensuring robust performance in detecting objects accurately. Furthermore, to assess the real-time performance of our proposed model, we evaluated the proposed method using several metrics such as latency, frames per second (FPS), throughput, and inference time. These metrics are crucial for assessing the efficiency and performance of DL models, particularly in applications requiring real-time or near-real-time processing [19,20].

### 2.4. Platform

The experiment works on Processor Intel(R) Core(™) i9-14900 K (32 CPUs) ~3.2 GHz 32 GB RAM and using NVIDIA A10 GPU (24 GB). All experiments were run on Windows 11 Pro 64 Bit, Python (Python 3.9.18) code using VS Code, Pytorch, Numpy, Pandas, ScikitLearn, Matplotlib, Seaborn, and Roboflow.

### 2.5. Ethics

This study received approval from the Health Research Ethics Committee of Central General Hospital Dr. Mohammad Hoesin Palembang, Indonesia, under ethical certificate No. 38/keprsmh/2021. The procedures adhered to the principles of the Declaration of Helsinki and International Ethical Guidelines for Biomedical Research Involving Human Subjects [22,23]. Written informed consent to participate in the study was obtained from the parents, legal guardian or next of kin of the participants. Detailed information about the examination procedures and objectives was provided to all research subjects. Subsequently, their participation in the research was sought through the signing of a consent form, indicating their agreement to take part. It is important to note that the research subjects participated voluntarily and retained the right to withdraw from the study at any point.

## 3. Result and Discussion

### 3.1. The Ablation Study

In this study, we conducted experiments on various Yolo architectures to identify the most effective model for CSD prediction. An ablation study focused exclusively on the Yolo model to detect and segment holes in each US video. Hence, the study did not include the testing of the complete framework (normal–abnormal classification, view classification, and defect detection). This research focuses on cardiac defects in children, which can be extremely small. This study aims to detect these small objects using various Yolo architectures, with a comprehensive comparison of the results. Such an architecture is a popular object detection model known for its speed and accuracy. However, Yolov1-v5 has historically struggled with detecting small objects, particularly in scenes with a high resolution. The grid-based approach divides the image, but when objects are smaller than the grid size, they can be easily missed [18,20]. For example, Yolov6-v7 struggles with detecting very small objects in images, especially when they are far from the camera or located in cluttered scenes. These models still face difficulty in localizing these objects accurately [20,21]. Yolov8 has improved in detecting smaller objects with its advanced feature extraction techniques, but small object detection remains challenging in very cluttered environments or when objects are very far from the camera [19,21]. In the ablation study, we compare three different Yolo architectures (Yolov5, Yolov7, and Yolov8) combined with five sizes ranging from nano to extra-large, resulting in a total of 12 variants, which were included in the ablation study. Yolov5 utilizes the CSPDarknet53 backbone while Yolov7 employs the Extended-ELAN backbone. Yolov8 utilizes the same backbone as Yolov7 but is enhanced with an anchor-free detector and multi-scale prediction to ensure real-time processing while maintaining the accuracy of small object detection. Yolov8 is a new state-of-the-art computer vision model built by Ultralytics (Frederick, MD, USA) [21], the creators of Yolov5. The Yolov8 model contains out-of-the-box support for object detection, classification, and segmentation tasks, accessible through a Python package as well as a command line interface. The models were trained with hyperparameters set to 50, 100, and 200 epochs. Validation was performed using batch sizes of 2 and 4, with the SGD optimizer configured at a learning rate of 0.001. This study compared different Yolo variants to evaluate which architecture or modifications most effectively enhanced model performance. The Yolo variants tested included Yolov5 (Yolov5n-Seg, Yolov5s-Seg, Yolov5m-Seg, Yolov5l-Seg, and Yolov5x-Seg), Yolov7 (Yolov7-Seg and Yolov7x-Seg), and Yolov8 (Yolov8n-Seg, Yolov8s-Seg, Yolov8m-Seg, Yolov8l-Seg, and Yolov8x-Seg). With 12 pre-trained models of Yolo variants available, 72 different models were produced after training with the US cardiac image dataset, which allowed for a comparative evaluation of the model performance in determining the optimal performance of each variant.

The performance of each model was assessed using several metrics: precision (B), recall (B), mAP50 (B), and mAP50–95 (B) for bounding box (B) detection, as well as precision (M), recall (M), mAP50 (M), and mAP50–95 (M) for mask (M) detection. It can be observed from the confusion matrix that Yolov8 outperformed the other models, both Yolov5 and Yolov7 (Figure 4). The performance metrics considered were derived from validation data and testing data (unseen). The mAP (B) reaches around 98% for the validation data and 72% for the unseen data, while the mAP (M) reaches around 71% for the validation data and 61% for the unseen data. For developing the Yolo model in this study, Yolov8 was selected. To identify the most suitable Yolov8 model for the proposed framework for detecting defects in pediatric cardiac US images, we evaluated all the variants under various conditions, including normal versus abnormal classification, five view classifications, and defect detection. A comprehensive experiment and a detailed comparison of the results are provided in the subsequent section with an in-depth analysis.

### 3.2. Normal–Abnormal Classification

In the ablation study, we found that the Yolov8 architecture outperformed both the Yolov5 and Yolov7 models. To enhance Yolov8’s performance in predicting CSDs, we implemented a stacking process to improve the hole detection results on the cardiac septum. The first step in this process involved classifying conditions as normal or abnormal. The Yolo classification process is different from that of traditional classification models. Instead of separately predicting bounding boxes and class probabilities, Yolo performs both tasks simultaneously in a single neural network architecture [21].

**Figure 5:**
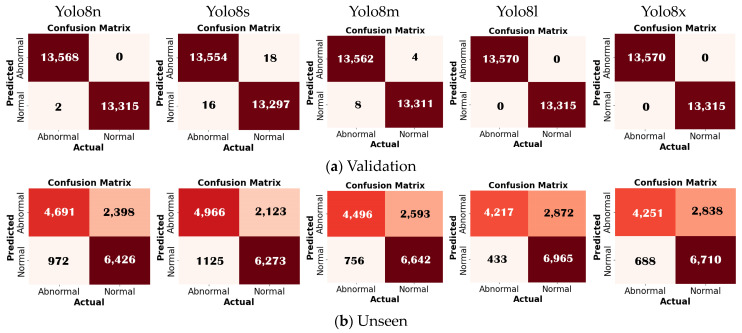
Normal–abnormal classification performance for five architectures.

**Figure 6:**
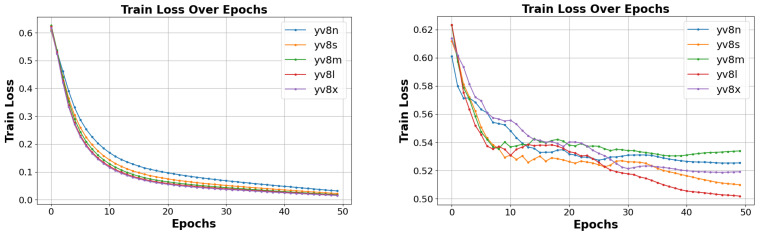
Normal and abnormal cardiac classification results in terms of training and validation loss.

We utilize frames extracted from US videos, with validation and test results conducted on unseen data. The confusion matrix aids in identifying incorrect frames. Among the five Yolo architectures tested, Yolov8l (large) demonstrates a superior performance. It achieved 100% accuracy, sensitivity, and specificity during training. However, when tested with unseen data, it achieved an accuracy of 77.19%, sensitivity of 90.69%, and specificity of 71.80% (Figure 5). The loss response for both the training and validation consistently highlighted YOLOv8l as the top performer among the architectures tested (Figure 6).

### 3.3. Standard View Classification

This study discusses the CSD classification using five standard echocardiographic views. This classification is usually performed to facilitate the analysis of the cardiac structure and function. Such a process is the essential first step in interpreting echocardiograms. These views include the subcostal views, apical views, and parasternal views, which are significant for the diagnosis of various ASDs, VSDs, and AVSDs. The YoloV8x reaches the best accuracy, sensitivity, and specificity performance for both the validation and unseen data in our classification results, as seen in Table 2. There was no significant decrease in the performance values between the training and testing processes, indicating that Yolov8l demonstrates the model’s suitability for performing view classification. Specifically, it effectively classifies views such as A4CH, A5CH, PLAX, PSAX, and SC.

**Figure 7:**
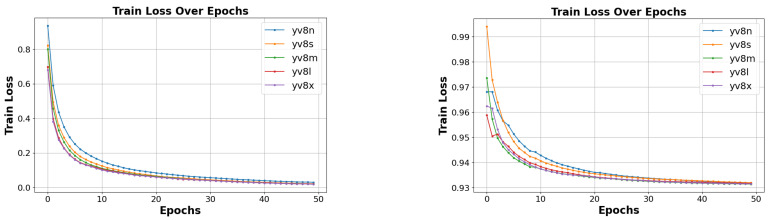
View classification result on training and validation loss.

In our model for view classification, the training loss measures how well the model fits the training data, while the validation loss evaluates its generalization to new, unseen data. Typically, the training loss decreases during training as the model learns to fit the training data (Figure 7). Conversely, the validation loss is crucial for detecting overfitting. Ideally, the validation loss should be as low as possible, often when the training loss is substantially lower. The stabilization of both losses at a specific point indicates an optimal fit.

### 3.4. Defect Detection

**Figure 8:**
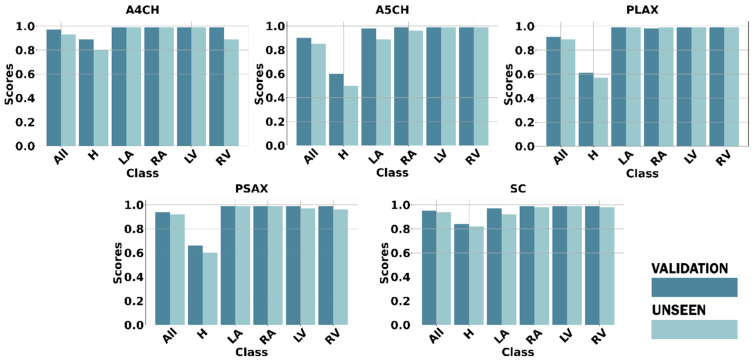
The CSD detection performance using our framework on five standard views.

Defect detection is the essential process of identifying and diagnosing anomalies within the heart. The timely detection of these anomalies is critical for initiating appropriate treatment and effectively managing CSD conditions, which can profoundly influence a child’s health and overall well-being. Based on the normal–abnormal and view classification results, we employed Yolov8l to perform defect detection. The mAP was utilized to assess the performance of both the validation and unseen data. We utilized the five standard cardiac views to ensure thorough results: A4CH, A5CH, PLAX, PSAX, and SC. These views play a pivotal role in precisely evaluating the cardiac chamber wall classes, including RA, RV, LA, LV, and identifying any septal defects (H) within the heart (Figure 8).

Chamber wall detection (LA, RA, LV, RV) across the five views yielded satisfactory results, both in terms of the validation and unseen data, with the mAP value exceeding 90%. However, in the defect detection or hole (H) detection, the mAP average value dropped below 85%. The A4CH and SC views successfully detect with a mAP of over 80%, whereas A5CH, PLAX, and PSAX only achieve mAP values below 65% for the validation data and below 60% for the unseen data. It can be observed that achieving accurate detection in both the A4CH and A5CH views poses significant challenges due to their similarity. The main point of differentiation in the A5CH view is the presence of the aorta in the middle of the chamber wall. As a result, errors are quite common during both the validation and unseen test phases. The parasternal view (PLAX and PSAX) provides a longitudinal slice of the heart, which may not offer a comprehensive view of all the cardiac structures. Certain defects, especially those located in less visible areas or those requiring a different angle of approach, may not be easily detected. However, achieving a mAP value greater than 50%, the model demonstrated the effective detection of cardiac defects.

Based on the test results obtained using the proposed framework, the model demonstrates a highly satisfactory performance in defect detection. Utilizing five views, we measured the mAP bounding box (BBox) and mAP pixel-wise binary mask (Mask) (Table 3). The mAP BBox quantifies the accuracy of object detection, while the mAP Mask evaluates the effectiveness of instance segmentation models. Both metrics are crucial for assessing the performance of the proposed Yolov8l. The results reveal that the achieved mAP ranges from 60% to 89% for the validation data and from 50% to 80% when tested with the unseen data. These findings indicate that the defect detection performance exceeds the baseline mAP threshold of 50%, indicating increased confidence levels in the detection outcomes.

### 3.5. CSD Decision-Making

**Figure 9:**
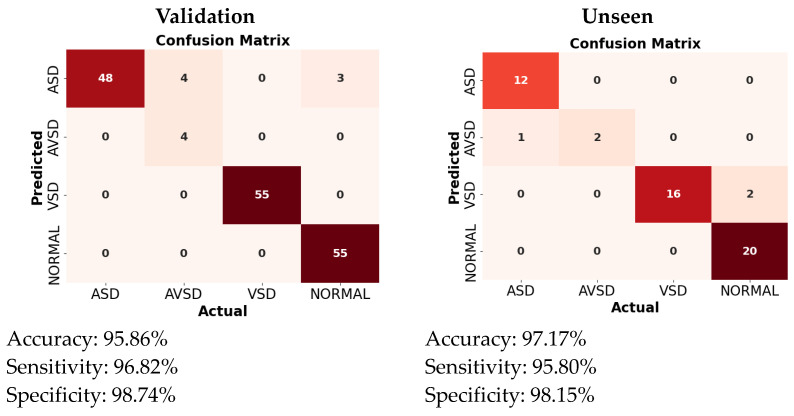
All CSD detection performances based on patient.

The outcomes of the model compared to actual US examinations by pediatric cardiology demonstrate a satisfactory performance (Figure 9). During the training process from 169 US videos, there were seven false detections in the ASD condition, while there were no errors in the VSD, AVSD, and normal conditions. The heart’s anatomy is complex, and ASDs can vary in size, shape, location, and associated anomalies. This variability makes it difficult for Yolo to accurately detect and classify defects without extensive training on diverse datasets. In some echocardiographic views, the area of interest (the atrial septum) might not be adequately captured, making it difficult for the model to identify the defect [3,6]. It can be observed, during the validation process, that the proposed model achieved an accuracy of 95.86%, sensitivity of 96.82%, and specificity of 98.74%. Meanwhile, during the testing process using the unseen data (US videos from patients not included in the training data), out of the 35 US videos, only three were diagnosed incorrectly. The accuracy was 97.17%, sensitivity was 95.80%, and specificity was 98.15%. From Figure 9, one of the incorrect predictions involved the AVSD condition. Due to the rarity of this condition, we had only seven US videos available—four for training and the remaining three for testing. The limited amount of training data resulted in insufficient learning, leading to one misprediction. Incorrect predictions of AVSDs can occur due to the complexities of medical imaging. AVSDs involve abnormalities in both the septum and the heart’s atrioventricular valves, which vary in size, shape, and severity across patients, making it harder for the model to generalize across different cases [24]. In addition, the tricuspid and mitral valves are often fused or malformed; detecting these subtle valve abnormalities is challenging for models trained primarily to detect septal defects, and the misidentification of valve issues can contribute to misprediction [24]. Moreover, there were 18 US videos of the perimembranous VSD condition in the testing process; however, 2 of them were incorrect predictions. Perimembranous VSDs are located near the tricuspid and aortic valves, and because of this, they may be obscured by the structures and motion of the valves, making them harder to detect in imaging studies, especially in 2D imaging techniques like standard echocardiograms [25]. Standard 2D echocardiograms, which are commonly used for diagnosis, may not always provide a clear view of the perimembranous area [17]. Despite this challenge, the model successfully detected all the unseen US videos depicting the normal, ASD, and VSD conditions without errors, indicating a robust performance overall. It is observed that the proposed model demonstrates a satisfactory performance, as it maintains a performance on the unseen test data close to that of the trained validation data. Therefore, it can be concluded that the proposed model effectively recognizes key features, such as holes, in the US images associated with the CSD condition.

To evaluate the proposed model under real-time conditions, we measured the latency, inference time, FPS, and throughput. The results in Table 4 highlight the model’s performance with and without GPU acceleration. When using a GPU, latency is significantly reduced to approximately 11.09 ms, demonstrating a very fast response time. However, the model takes around 13.69 s to complete a single inference due to the complexity of the background in the real-time processing of the US CSDs videos. Despite this, the system achieves a processing speed of 90.38 frames per second, allowing it to render or process over 90 frames each second. Based on this performance, it can be concluded that the proposed model is suitable for use in a real-time application, achieving a throughput of 249.20 frames every 5 s.

### 3.6. Benchmarking with State of the Art

Our proposed end-to-end real time framework was benchmarked against two previous studies which was a considered a fair experiment, because they have a complete framework for classifying CSD conditions (Table 5). However, because these studies are very limited, both only use cases of ASDs. Lin et al. [3] proposed a 3D-UNet architecture for automatic ASD detection that is applicable to color Doppler echocardiography. They suggested the four standard views for ASD detection and quantification, i.e., 4cv, PSAX, subxiphoid sagittal view (SC2A), and subxiphoid 4CV (SC4CV). Regarding the results, they obtained an average accuracy of 99% in four standard views for ASD detection and identification. Hong et al. [4] proposed a DL architecture with ResNeSt-200 for ASD detection based on the subcostal atrium septum (subAS), 4CV, the low parasternal four-chamber (LPS4C), and PSAX views. They yielded an average accuracy of 99.42%, 91.26% sensitivity, and 99.83% specificity in four standard views. Unlike the method we employed, although still utilizing the five standard views, the detected CSDs were comprehensive, specifically those of ASDs, VSDs, and AVSDs. Based on the results of the proposed model, it displays a 95.86% accuracy, 96.82% sensitivity, and 98.74% specificity. Our findings surpass those of [3] and closely align with the outcomes reported in [4]. However, it is worth noting that both [3,4] exclusively target one category of CSDs, whereas our study extends its scope to detect three distinct classes. Moreover, our research includes rigorous testing with real-time patient data sourced directly from hospital records, underscoring the robustness and real-world applicability of our approach.

### 3.7. The CSD Visualization

**Figure 10:**
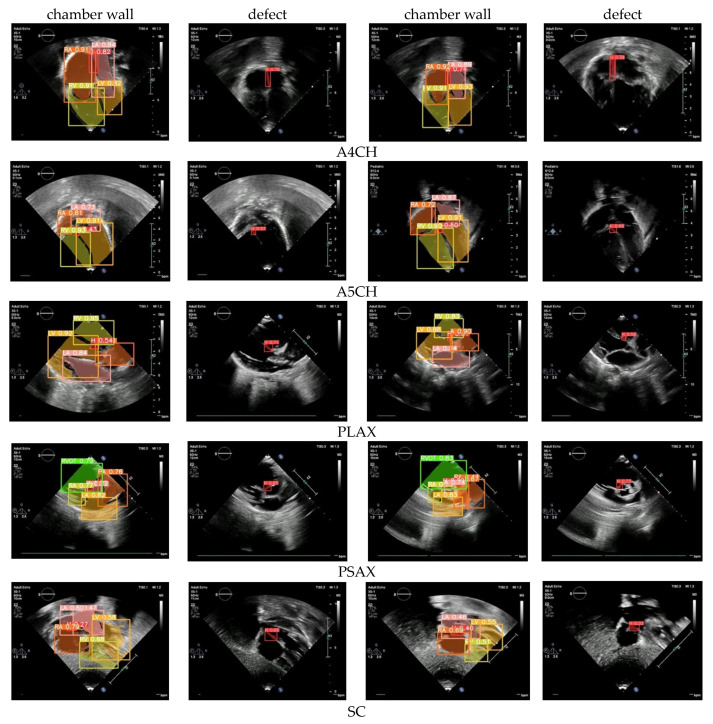
The sample of CSD detection images in five views.

Detecting defects can be challenging due to the heart being a complex three-dimensional organ with intricate structures. Some defects may be subtle or located in areas where they are obscured by other cardiac structures, making them difficult to identify from a single imaging plane. Our framework uses a combination of five views to make a CSD decision. A visualization of our results to show an object detection scheme on the chamber wall and hole is shown in Figure 10. There are walls that separate the chambers, and each chamber has its own walls. The septum is the primary wall that divides the cardiac into left and right sides. There is the interatrial septum between the atria and the interventricular septum between the ventricles. LA, LV, RA, RV are objects that must be detected on the chamber wall, so as to assist in determining the position of defects or holes. By identifying the position of defects or holes in these walls, it helps in diagnosing the type of CSD.

The value obtained in each image represents the confidence level of the detection results. For the purposes of this study, a confidence value exceeding 0.5 can be deemed indicative of a reliable decision. It can be observed that all the confidence values are above the baseline value, meaning that the proposed Yolov8l model is capable of exhibiting a reliable detection performance for defects in the cardiac septum.

### 3.8. Color Doppler Echocardiography Case

**Figure 11:**
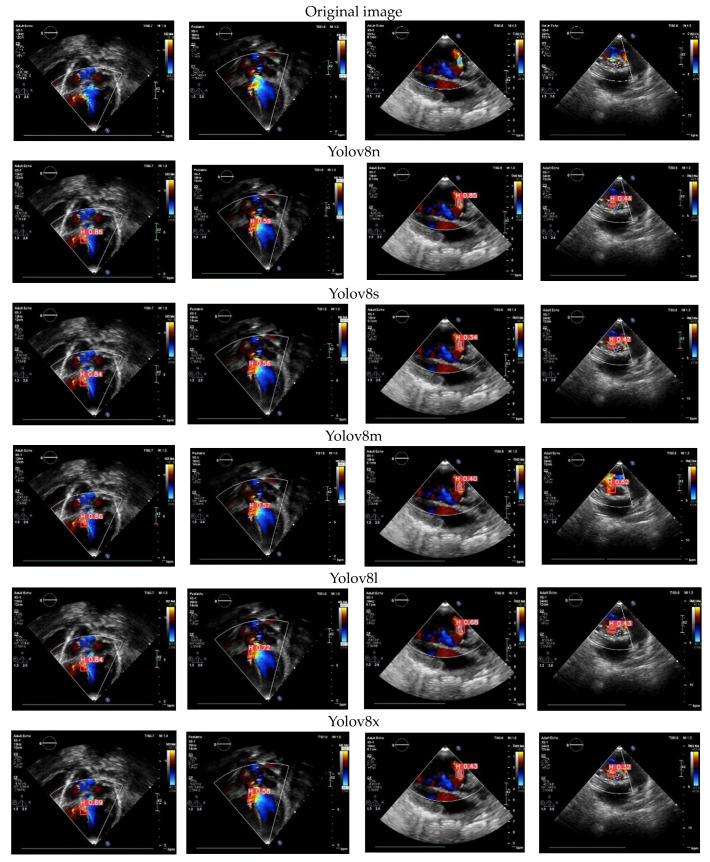
The proposed model of CSD detection in color Doppler echocardiography case.

In this study, we tested the proposed model using color Doppler echocardiography. The predicted outcomes for the cardiac defects are visualized in Figure 11, displaying the results from five Yolov8 architectures. These models are trained to identify specific views required for evaluating CSDs and detect the presence of cardiac defects. In this depiction, the US video utilizes red to represent blood flow toward the transducer and blue to signify blood flow away from the transducer [4]. This capability enables our proposed model of cardiac defect detection to be available not only for the original echocardiography but also with the additional insights provided by color Doppler echocardiography. Among the five proposed architectures, Yolov8l consistently delivers a satisfactory performance and effectively detects holes in the septum. The resulting confidence values exhibit considerable variability, spanning from 0.84 to 0.43. This range of confidence values signifies the model’s adeptness in detecting CSDs through color Doppler echocardiography.

Utilizing DL in color Doppler echocardiography for detecting CSDs offers numerous advantages [3,4]. It streamlines the evaluation of echocardiographic videos, a typically time-intensive process necessitating expert clinical skills. DL can automate this task, saving valuable time and resources. Furthermore, DL models are capable of discerning intricate patterns within echocardiographic images that may elude human experts, enhancing the potential for precise diagnoses [5]. This integration of DL technology holds promise for improving the efficiency and accuracy of CSD detection in clinical settings.

### 3.9. Limitations

While this research demonstrates good performance, future developments should take into account the following considerations: (i) The dataset utilized did not contain information on defect sizes, resulting in the detection model not accounting for the various types of cardiac septal defects. It would be beneficial for defect sizes to be incorporated as one of the parameters in decision-making. (ii) The data were limited by the study’s retrospective nature, and the study included a relatively small number of patients. Although our model achieved good performance in the external test set, testing the model in a prospective multi-center cohort is warranted. (iii) In this study, only three types of defects were utilized: secundum ASDs, outlet perimembranous VSDs, and complete/incomplete AVSDs. Future research will encompass additional cases like primum ASDs, sinus venosus ASDs, doubly committed VSDs, and muscular VSDs to generalize the framework for all types of CSDs. In conclusion, although the current prototype exhibits promise, continuous research and development play a vital role in refining the proposed model, addressing its limitations, and ensuring its relevance across a broader spectrum of scenarios within the realm of pediatric cardiology health.

## 4. Conclusions

Our study introduces an innovative real-time framework tailored for pediatric US video analysis to enhance the diagnosis of CSDs. By integrating DL techniques, specifically leveraging the Yolo architecture, our model achieves a high accuracy and robust performance in CSD decision-making, with satisfactory performance. Notably, the Yolov8l model demonstrates a precise real-time performance, as evidenced by testing on US video sourced directly from Dr. Mohammad Hoesin General Hospital in Palembang, Indonesia. During the validation phase, the proposed model achieved an accuracy of 95.86%, sensitivity of 96.82%, and specificity of 98.74%. When tested with unseen data, an accuracy of 97.17%, sensitivity of 95.80%, and specificity of 98.15%. With only 3 misdiagnoses out of 35 US videos, this comprehensive approach shows strong potential to streamline clinical decision-making and improve patient outcomes in pediatric cardiology. This framework represents a significant advancement in the digitization of echocardiography, promising enhanced efficiency and accuracy in diagnosing CSDs, ultimately benefiting pediatric patients and healthcare practitioners alike.

## Figures and Tables

**Table 1:** The total utilized US video and US images.

Class	Data	Total US Videos	Total US Images
Normal	Training	61	53,256
	Validation		13,315
	Real-time testing	14	8500
	(unseen data)		
Abnormal	Training	126	54,276
(ASD, VSD, and AVSD)	Validation		13,570
	Real-time testing	21	8189
	(unseen data)		
Total		222	151,106

**Table 2:** The view classification performance for five architectures.

Architecture	Accuracy (%)		Sensitivity (%)		Specificity (%)	
	Validation	Unseen	Validation	Unseen	Validation	Unseen
YoloV8n	97.45	90.74	97.32	89.72	99.36	97.74
YoloV8s	97.41	90.84	97.27	89.23	99.35	97.74
YoloV8m	97.50	90.22	97.37	88.76	99.37	97.59
YoloV8l	97.45	92.26	97.33	91.22	99.36	98.11
YoloV8x	97.50	91.50	97.37	90.45	99.37	97.92

**Table 3:** Cardiac defect detection performance on mAP(50).

View	Validation (mAP)		Unseen (mAP)	
	BBox	Mask	BBox	Mask
A4CH	0.89	0.79	0.80	0.74
A5CH	0.60	0.74	0.50	0.61
PLAX	0.61	0.83	0.57	0.56
PSAX	0.66	0.62	0.60	0.60
SC	0.84	0.78	0.82	0.76

**Table 4:** The real-time processing of the proposed model.

Only CPU			With GPU		
Latency (ms)	Inference time (ms)	FPS (Hz)	Latency (ms)	Inference time (ms)	FPS (Hz)
67.45	29,597.06	15.31	11.09	13,693.35	90.38

**Table 5:** Benchmarking previous studies of CSD detection.

Authors	CSDs	DL Architecture	Accuracy (%)	Sensitivity (%)	Specificity (%)
Lin et al. [3]	ASD	3D-UNet	99	88	89
Hong et al. [4]	ASD	ResNeSt-200	99.42	91.26	99.83
Our model (validation)	ASD, VSD, AVSD	Yolov8l	95.86	96.82	98.74
Real-time testing			97.17	95.80	98.15

## Data Availability

The datasets generated and/or analyzed in this study are available in https://github.com/ISySRGg/Echo-Infant (accessed on 20 July 2023).
